# Dynamics of multimorbidity, health expectancy, and survival in middle aged and older individuals

**DOI:** 10.1093/gerona/glaf164

**Published:** 2025-07-29

**Authors:** Elisa Fabbri, Julián Candia, Toshiko Tanaka, Ann Zenobia Moore, Paolo Muratori, Agar Brugiavini, Amaia Calderón-Larrañaga, Davide L Vetrano, Laura Fratiglioni, Eileen Crimmins, Jessica Faul, Kenneth M Langa, David Weir, Luigi Ferrucci

**Affiliations:** Division of Internal Medicine, Morgagni-Pierantoni Hospital, Forlì, Italy; Department of Medical and Surgical Sciences, University of Bologna, Bologna, Italy; Longitudinal Studies Section, Translational Gerontology Branch, Intramural Research Program, National Institute on Aging, National Institutes of Health, Baltimore, Maryland, United States; Longitudinal Studies Section, Translational Gerontology Branch, Intramural Research Program, National Institute on Aging, National Institutes of Health, Baltimore, Maryland, United States; Longitudinal Studies Section, Translational Gerontology Branch, Intramural Research Program, National Institute on Aging, National Institutes of Health, Baltimore, Maryland, United States; Division of Internal Medicine, Morgagni-Pierantoni Hospital, Forlì, Italy; Department for Life Quality Studies, University of Bologna, Bologna, Italy; Department of Economics, University of Venice, Venice, Italy; Aging Research Center, Department of Neurobiology, Care Sciences and Society, Karolinska Institutet and Stockholm University, Stockholm, Sweden; Stockholm Gerontology Research Center, Stockholm, Sweden; Aging Research Center, Department of Neurobiology, Care Sciences and Society, Karolinska Institutet and Stockholm University, Stockholm, Sweden; Stockholm Gerontology Research Center, Stockholm, Sweden; Aging Research Center, Department of Neurobiology, Care Sciences and Society, Karolinska Institutet and Stockholm University, Stockholm, Sweden; Stockholm Gerontology Research Center, Stockholm, Sweden; Leonard Davis School of Gerontology, University of Southern California, Los Angeles, California, United States; Institute for Social Research, University of Michigan, Ann Arbor, Michigan, United States; Institute for Social Research, University of Michigan, Ann Arbor, Michigan, United States; Department of Internal Medicine, University of Michigan, Ann Arbor, Michigan, United States; Institute for Social Research, University of Michigan, Ann Arbor, Michigan, United States; Longitudinal Studies Section, Translational Gerontology Branch, Intramural Research Program, National Institute on Aging, National Institutes of Health, Baltimore, Maryland, United States

**Keywords:** Aging, Multimorbidity, Health expectancy, Survival, Mortality

## Abstract

**Background:**

Life expectancy has increased, but such increase has disproportionally expanded the period of life with diseases. Whether expanding health expectancy (HE), defined as years of life free of chronic diseases, could also affect rate of multimorbidity accumulation is uncertain. Objective: to investigate the dynamic relationship between HE and rate of multimorbidity accumulation and their impact on survival.

**Methods:**

Four thousand two hundred seventy-four (3511 > 50 years) participants from the Health and Retirement Study (HRS), healthy at baseline and developing at least one disease overtime were included. Mean baseline age was 55.1 years and average follow-up was 9.4 years. Multimorbidity was operationalized as count of diagnosed diseases from a list of nine chronic conditions. HE was operationalized as years from birth until when the first disease was ascertained, and percentage of life in good health calculated as percentage of life lived free of chronic diseases. Mixed models investigated the association between HE and rate of multimorbidity accumulation, while survival analyses evaluated association with time to death.

**Results:**

HE were positively associated with multimorbidity rate (*P* < .001). Shorter HE and faster multimorbidity rate were independently associated with higher mortality (*P* < .001). Their interaction was negatively associated with mortality (*P* < .001). Results were confirmed restricting the analysis to individuals 51 or older and using HRS specific weights. Individuals with longer HE experienced a greater survival, almost regardless of multimorbidity rate, while a positive gradient was found in percentage of life in good health linked to multimorbidity rate.

**Conclusions:**

Expanding health expectancy is likely followed by compression of morbidity.

## Introduction

With the aging of the population, the number of individuals affected by multiple chronic diseases is rapidly increasing, posing significant challenges to healthcare, social, and political systems that have predominantly been structured under the “one patient, one disease” paradigm.^1^ Multimorbidity, which has been conceptualized as the concurrent presence of multiple chronic diseases within a single individual,^1-4^ is the currently most common health condition and its epidemiological burden is expected to further increase in the future.^5^ Historically, medicine has tried to sort diseases into more and more specific groups to better understand which treatment works best. However, emerging evidence indicates that many age-related chronic diseases, particularly those more commonly observed in older adults, share common pathophysiological mechanisms and risk factors.^6^^,^^7^ These mechanisms include the impairment of biological processes that prevent the accumulation of age-related damage, such as mitochondrial oxidative phosphorylation, proteostasis, and DNA repair, which are linked to biological aging.^8-11^ A current hypothesis suggests that the rate of accumulation of multimorbidity is a consequence of and may serve as a proxy for the pace of biological aging.^12^ Specifically, individuals who accumulate diseases faster than others are experiencing accelerated aging. The accumulation of damage in macromolecules and organelles, coupled with diminished resilience mechanisms, can manifest clinically as reduced organ function and loss of physiological reserves. This often presents initially as an impaired response to stressors before evolving into recognizable clinical entities. Supporting this hypothesis, disease diagnoses are often preceded by a prolonged period when underlying pathology causes no symptoms or only mild symptoms.

With few exceptions, the medical system primarily intervenes in diseases once they become clinically manifest. Rapid advancements in medical and surgical treatments have significantly enhanced survival rates and quality of life for patients with multiple diseases.^13^ However, whether health expectancy, the portion of life spent free of clinically evident diseases, has also increased proportionally is doubtful. Thus, individuals live longer but experience poor health for a larger percentage of their lives because the duration of life lived with chronic diseases or frailty has disproportionally increased.^14-16^ This shift has increased the burden of multimorbidity on the overall population.

To counteract this trend, it is crucial to focus on promoting longer health expectancy through behavioral and pharmacological preventive interventions aimed at decelerating the aging process, thereby delaying the onset of multiple chronic diseases and reducing the rate of multimorbidity accumulation. However, it is still uncertain whether interventions that improve health expectancy also effectively reduce the rate at which diseases accumulate, and whether increased health expectancy results in a larger proportion of overall lifespan lived free of chronic diseases. While answering these questions is challenging short of performing a population-­wide intervention, we can start addressing it by examining the relationship between health expectancy, the accumulation of diseases, and subsequent survival. According to the geroscience hypothesis, we can anticipate that slower biological aging would be associated with longer health expectancy, reduced rate of accumulation of morbidity, and longer life. However, to the best of our knowledge, the interplay between health expectancy, the rate of multimorbidity accumulation, and longevity has not been jointly examined before.

One of the obstacles to the study of health expectancy is the lack of a widely accepted operational definition for it.^17^ In this study, we operationalize health expectancy at the individual level as “years of life free of chronic diseases” assessed as years from birth until the first chronic disease is ascertained. Leveraging data from the Health and Retirement Study (HRS), our research aims to investigate the relationship between the years of life free of chronic diseases and the longitudinal accumulation of additional diseases afterward. We also explored the independent and potentially synergistic impact of both health expectancy and rate of disease accumulation on survival.

## Methods

### Study population

The Health and Retirement Study (HRS) is a nationally representative longitudinal and multidisciplinary survey of individuals aged 51 years or older and their spouses in the United States that began in 1992. The HRS is sponsored by the National Institute on Aging (grant number NIA U01AG009740) and is conducted by the University of Michigan. A detailed description of the study has been published previously.^18^ This analysis used data from the 1992-2020 RAND HRS Longitudinal File.^19^^,^^20^ This file was developed at RAND with funding from the National Institute on Aging and the Social Security Administration. Moreover, data from the 1992-2020 RAND HRS Longitudinal File were merged with data from the HRS Cross-Wave Tracker File,^21^ which contains information about vital status.

### Sample selection

We analyzed data from three HRS cohorts, specifically the Original HRS (born 1931–1941), War Babies (born 1942-1947), and Early Baby Boomers (born 1948-1953). These cohorts enrolled subjects in their middle age and their spouses and followed them through older ages as shown in Figure S1 (see online supplementary material for a color version of this figure). All the participants from the three cohorts with available data on multimorbidity at baseline and follow-up visits were eligible for inclusion (*N* = 19 675). To estimate the years of life free of chronic diseases from birth up to the first visit when at least one chronic condition was diagnosed, we selected participants who were healthy (free of chronic diseases) at baseline and developed at least one disease during the follow-up period (*N* = 4274). Since the HRS is a nationally representative study for subjects aged 51 or older, we further selected a subsample of individuals older than 50 years (*N* = 3511). The flow chart detailing the selection process for the final study sample can be found in Figure S2 (see online Supplementary material for a color version of this figure).

### Multimorbidity

Multimorbidity is widely understood as the coexistence of multiple chronic conditions.^2^^,^^3^ However, numerous and heterogeneous tools or indexes have been proposed as metrics of multimorbidity.^22-24^ Such methodological heterogeneity reflects not only a tremendous clinical heterogeneity among individuals with multiple chronic conditions, in terms of number and combinations of conditions, severity of illness, and functional limitations^25^ but also a large heterogeneity among studies in the type, structure, and source of information available to code for multimorbidity, ranging from self-reported information to electronic medical records. Moreover, although multimorbidity has been traditionally operationalized as counts or weighted indices of chronic conditions,^4^ different analytic approaches have been attempted in relation to specific research purposes.^26^^,^^27^ For example, over the past decade, a large number of studies has been conducted to identify clusters or patterns of chronic conditions that co-occur more frequently than by chance and to investigate how these clusters or patterns evolve longitudinally, to understand possible underlying mechanisms and to develop strategies for primary, secondary and tertiary prevention.^4^^,^^28^^,^^29^ More recently, also the sequence of acquisition of specific subsets of diseases has been investigated to better characterize the temporal progression and chronological expansion of multimorbidity.^30^ Despite the large amount of literature on this topic, a universally accepted operational definition for multimorbidity is still lacking and none of the proposed definitions is widely considered as the gold standard. For the current exploratory analysis, which was aimed at investigating the dynamics of multimorbidity in relation to the duration of health expectancy, multimorbidity was operationalized as the count of diagnosed diseases from a predefined list of nine candidate chronic conditions. These conditions were selected a priori and were assessed longitudinally in the Health and Retirement Study (HRS).

Information on chronic diseases in HRS includes seven self-reported physician-diagnosed diseases that are common among older adults, assessed at each study wave: Hypertension, Diabetes, Heart Disease (including heart attack, coronary heart disease, angina, congestive heart failure, or other heart problems), Chronic Lung Disease (including chronic bronchitis or emphysema and excluding asthma), Cancer (including any malignant cancer except skin cancer), Stroke (including stroke or transient ischemic attack) and Arthritis. Participants were asked, “Has a doctor ever told you that you have…?” Additionally, we assessed the status of Cognitive Impairment and Depression, both of which are highly prevalent among older adults and are strongly linked to negative outcomes such as functional impairment, disability, and increased mortality. Cognitive Impairment was defined as being cognitively impaired or having dementia according to the Langa–Weir Classification.^31^ Finally, Depression was defined as reporting four or more depressive symptoms using the 8-item Center for Epidemiologic Studies Depression Scale (CES-D).^32^

### Health expectancy and survival

Health Expectancy was operationalized as “years of life free of chronic diseases,” assessed as years between birth and the first visit when at least one disease from the list mentioned above was first ascertained. Specifically, self-reported health conditions were based on self-reported diagnoses given by a physician since the last interview, while depression and dementia were assessed using specific neuropsychological tests conducted at the time of the interview.

The survival status in HRS has been ascertained through two primary sources: (1) HRS tracking information and (2) National Death Index linkage. A previous analysis of HRS data from 1992 to 2011 demonstrated high concordance between the two sources and a comparison with life tables confirmed that mortality ascertainment in the HRS was effectively complete.^33^ Information about survival status was available up to November 2024.

### Statistical analyses

Baseline characteristics of the study population are presented as means ± standard deviations (*SD*), medians with interquartile ranges (IQR), or percentages.

For the main analysis, we selected individuals who were healthy at baseline and subsequently developed at least one chronic disease during the follow-up period (*n* = 4274). For each participant, we calculated the years of life free of chronic diseases. The pace of accumulation of subsequent diseases following the first onset (measured as the number of new diseases per year) was estimated by mixed models.

To explore the relationship between years of life free of chronic disease and the rate of multimorbidity accumulation, we initially conducted Spearman correlation analyses. We then fit linear mixed models to assess whether individuals who developed their first chronic disease at older ages experienced a significantly different rate of multimorbidity accumulation compared to those who developed their first disease at younger ages. These analyses were first adjusted for sex, and then, further adjusted for additional covariates, including ethnicity, education, and time-varying BMI. Specifically, we adjusted for time-varying BMI because previous studies show a secular trend in obesity so people who were captured in different time periods may have different BMI systematically.^34^ Moreover, analyses were replicated by restricting the sample to individuals over 50-year old at study entry as well as using HRS-specific person-level analysis weights.

**Figure 1.**
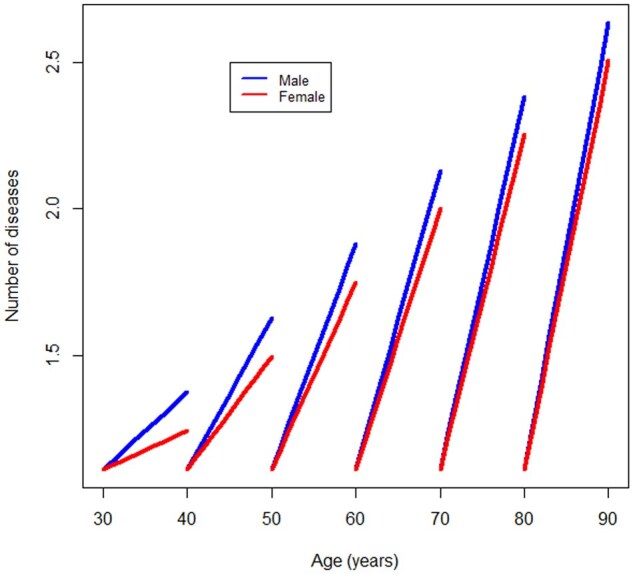
Longitudinal trajectories of multimorbidity accumulation after the end of the disease-free period of life according to different ages at onset of the first chronic disease in men (blue line) and women (red line) (*n* = 4274).

Longitudinal trajectories of multimorbidity accumulation were also assessed in men and women separately and for different groups of age at onset of the first disease (Figure 1).

**Figure 2.**
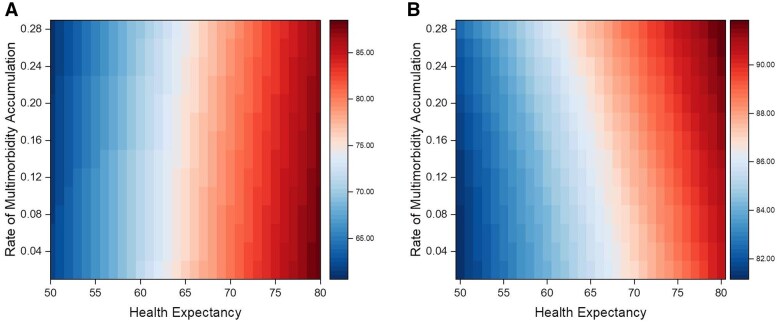
Heat map representing survival (A) and percentage of life in good health (B) according to health expectancy (= years of life free of chronic diseases), rate of multimorbidity accumulation and their interaction. (A) Represents survival from 60 (dark blue) to 90 (dark red) years according to health expectancy (*x*-axis) and rate of multimorbidity accumulation (*y*-axis). (B) Represents percentage of life in good health (from 80% to 92%) according to health expectancy (*x*-axis) and rate of multimorbidity accumulation (*y*-axis).

Moreover, we employed survival analyses to investigate the independent effects of health expectancy and the rate of multimorbidity accumulation, along with their interaction, on survival. Kaplan–Meier curves were generated, and log-rank tests were used to compare survival curves across different tertiles of age at onset of the first disease and across tertiles of the rate of multimorbidity accumulation. Additionally, hazard ratios (HR) were estimated in cox proportional hazard ratio regression models testing the association between years of life free of chronic diseases, rate of multimorbidity accumulation, and their interaction “years of life free of chronic diseases * rate of accumulation” with mortality. Additionally, for each combination of the age at which the first disease occurred (ranging from 50 to 80 years) and the rate of multimorbidity accumulation (ranging from 0.02 to 0.28 diseases per year), we estimated survival time (number of years from birth until death or until the last follow-up date when the participant was still alive) and calculated the percentage of life in good health (years of life free of chronic diseases divided by survival time, multiplied by 100). These results are presented in heat maps (Figure 2A and B).

Finally, we questioned whether selecting participants with no diseases at baseline may affect the generalizability of our results and whether the rate of multimorbidity accumulation was similar in individuals with different numbers of diseases at baseline. Therefore, in an additional analysis, we examined the longitudinal trajectories of multimorbidity accumulation among participants from the three HRS cohorts who reported zero (*n* = 5593), one (*n* = 6054), or two (*n* = 4127) chronic diseases at baseline. Using mixed models, we estimated the slope of the transition from 0 to 1 disease, from 1 to 2 diseases, and from 2 to 3 diseases for each group, respectively. The purpose of this analysis was to determine whether multimorbidity accumulation rates were similar among participants who, at the first HRS evaluation, had zero, one, or two diseases. We hypothesized that the rate of multimorbidity accumulation (= the slope of the trajectories) would be similar regardless of the number of diseases at baseline.

All analyses were performed using the SAS statistical package, version 9.4 (SAS institute Inc., Cary, NC) and R 4.3.1.

## Results

### Sample population

The eligible sample population included 19 675 individuals. For our research, we selected 4274 individuals who were healthy at baseline and developed at least one chronic disease during the follow-up period. The main characteristics of this sample are summarized in Table S1. Among these participants, 1849 (43.3%) were men. The average baseline age was 55.1 years (±6.3), with a range from 23 to 84 years. Three thousand, five hundred eleven individuals (82.1%) were 51 years or older. Ethnic distribution included 3536 White (82.8%) and 480 Black (11.2%) participants. The average baseline BMI was 26.5 kg/m^2^ (±5.6). Average education was 13 years [interquartile range: 12-16] and the average follow-up was 9.4 years (±5.4).

### Health expectancy and multimorbidity accumulation

We used mixed models on the final population sample (individuals with no diseases at baseline and who developed at least one disease over the follow-up, *N* = 4274) to estimate the rate of accumulation of additional diseases (slope) after the development of the first disease.

Overall, the average age at onset of the first chronic disease was 61.3 (±7.6) years, while the average rate of accumulation of additional diseases after the onset of the first disease was 0.1 diseases per year (ie, 1 disease/10 years; Figure S3, see online Supplementary material for a color version of this figure). Moreover, on average, men developed the first disease at a slightly but significantly older age than women (62.9 ± 7.3 vs 60.1 ± 7.6 years, *P* < .001) and accumulated additional diseases slightly faster than women (0.10 vs 0.09 diseases per year, *P* < .001).

We explored the relationship between years of life free of chronic diseases and the rate of subsequent accumulation of additional diseases. We found a positive correlation between the years free of chronic diseases and the rate of multimorbidity accumulation following the onset of the first chronic disease (Spearman Correlation: *r* = 0.1817, *P* < .001; Figure S4, see online Supplementary material for a color version of this figure). Similar results were also found by restricting the analysis to individuals over 50-year old at the study entry (*r* = 0.1552, *P* < .001) as well as using HRS-specific person-level analysis weights (*r* = 0.1814, *P* < .001). These findings suggest that individuals who developed their first chronic disease at older ages experienced a faster accumulation of additional diseases. Stratified analyses yielded similar results for both men (Spearman Correlation: *r* = 0.16, *P* < .001) and women (Spearman Correlation: *r* = 0.17, *P* < .001). These results were robustly confirmed using mixed-effects models, with health expectancy, operationalized as years of life free of chronic diseases, as the independent variable and the rate of multimorbidity accumulation as the dependent variable. These analyses were adjusted for sex, ethnicity, education, and time-varying BMI and results were confirmed by restricting the analysis to individuals over 50-year old at study entry as well as using HRS-specific person-level analysis weights (Table 1). Consistent results were observed in both men and women, and the interaction term for sex, age at first onset, and time was not significant. Figure 1 depicts the longitudinal trajectories of multimorbidity accumulation after the onset of the first chronic disease for both men and women according to different age groups at first onset. The plot clearly illustrates that the rate of multimorbidity accumulation was significantly faster in individuals who developed their first morbidity at older ages. Notably, we observed that men accumulated additional diseases at a significantly faster rate than women; however, there were no significant interactions between sex and age at the onset of the first chronic disease.

**Table 1.** Health expectancy (=years of life free of chronic diseases) and longitudinal increase of the number of chronic diseases over the follow-up after the development of the first disease.

	Number of chronic diseases		
	Model 1 (*N* = 4274)	Model 2 (*N* = 3511 aged 50+)	Model 3 (using HRS weights)
**Health expectancy, years**	0.004 (0.001)***	0.005 (0.002)**	0.004 (0.001)**
**Sex (women vs men)**	−0.07 (0.019)***	−0.09 (0.021)***	−0.07 (0.020)***
**Time**	0.094 (0.002)***	0.099 (0.001)***	0.099 (0.0003)***
**Health expectancy × time**	0.002 (0.0003)***	0.003 (0.0003)***	0.002 (0.0003)***
**Sex (women vs men) × time**	−0.012 (0.004)***	−0.011 (0.004)**	−0.015 (0.004)***

This analysis was performed in the whole sample population (model 1), in participants older than 50 years only (model 2), and using HRS-specific person-level analysis weights (model 3). Results from linear mixed models adjusted for ethnicity, education and time-varying BMI.

* *P* < .05.

** *P* < .01.

*** *P* < .001.

### Health expectancy, multimorbidity accumulation, and survival

In subsequent analyses, we investigated how the years of life free of chronic diseases and the rate of multimorbidity accumulation relate to survival. Kaplan–Meier survival curves were first plotted according to different tertiles of age at onset of the first chronic disease (Figure S5, see online Supplementary material for a color version of this figure) and then by tertiles of rate of multimorbidity accumulation (Figure S6, see online Supplementary material for a color version of this figure). We found that more years free of chronic diseases were associated with longer survival (log-rank test: *P* < .001), while a faster rate of multimorbidity accumulation after the end of health expectancy was associated with shorter survival (log-rank test: *P* < .001).

In further analyses, we used Cox proportional hazard regression models to explore the relationship of health expectancy, rate of disease accumulation, and their interaction with survival (Table 2). This analysis confirmed that fewer years free of chronic diseases and a faster rate of multimorbidity accumulation were significantly and independently associated with mortality (*P* < .001). Again, the result was confirmed by restricting the analysis to individuals over 50-year old at study entry as well as using HRS-specific person-level analysis weights. Additionally, we identified a significant interaction between the years of life free of chronic diseases and the rate of multimorbidity accumulation, which was negatively associated with mortality (*P* < .001). In participants with longer life free of chronic diseases, the hazard ratios for each additional unit of disease accumulation were lower than in those with shorter life free of chronic diseases. Overall, these data suggest that a longer healthy life experience a faster deterioration of health that is not fully explained by chronic diseases accumulation.

**Table 2.** Health expectancy (=years of life free of chronic diseases), rate of multimorbidity accumulation and mortality.

	Mortality					
	Model 1 (*N* = 4274)		Model 2 (*N* = 3511 aged 50+)		Model 3 (using HRS weights)	
	*β* (*SE*)	HR	*β* (*SE*)	HR	*β* (*SE*)	HR
**Health expectancy (years)**	−0.06 (0.005)***	0.94	−0.05 (0.006)***	0.95	−0.05 (0.005)***	0.94
**Rate of multimorbidity accumulation (*n* diseases/year)**	5.50 (0.41)***	245.73	5.08 (0.43)***	161.48	5.48 (0.41)***	240.5

	Model 1 (*N* = 4274)	Model 2 (*N* = 3511 aged 50+)	Model 3 (using HRS weights)
	*β* (*SE*)	*β* (*SE*)	*β* (*SE*)
**Health expectancy (years)**	−0.03 (0.009)***	−0.03 (0.01)**	−0.03 (0.009)***
**Rate of multimorbidity accumulation (*n* diseases/year)**	19.25 (3.76)***	14.54 (4.64)**	19.50 (3.73)***
**Health expectancy × rate of accumulation (interaction term)**	−0.21 (0.06)***	−0.14 (0.07)*	−0.22 (0.06)***

This analysis was performed in the whole sample population (model 1), in participants older than 50 years only (model 2) and using HRS-specific person-level analysis weights (model 3). Cox proportional hazards model.

* *P* < .05.

** *P* < .01.

*** *P* < .001.

While individuals with longer health expectancy generally live longer, it remains unclear whether the percentage of their lives spent without disease is longer, similar, or shorter than that of individuals with shorter health expectancy. To investigate this question, we estimated survival time and calculated the percentage of life in good health (years of life free of chronic diseases divided by survival time, multiplied by 100) for each combination of years of life free of diseases (ranging from 50 to 80 years) and rate of disease accumulation (ranging from 0.02 to 0.28 diseases per year). The results are presented in Figure 2 using heat maps. More years free of chronic diseases were associated with both longer survival and a higher percentage of life in good health. Specifically, individuals with longer health expectancy tended to experience a greater survival, almost regardless of the rate of multimorbidity accumulation, while there was a positive gradient in the percentage of life in good health linked to the rate of multimorbidity accumulation (Figure 2A and B). In conclusion, individuals who experienced more years free of chronic diseases and a faster rate of multimorbidity accumulation after the first disease diagnosis were those with the highest percentage of life in good health.

### Additional analysis: trajectories of multimorbidity accumulation according to the number of diseases at baseline

**Figure 3.**
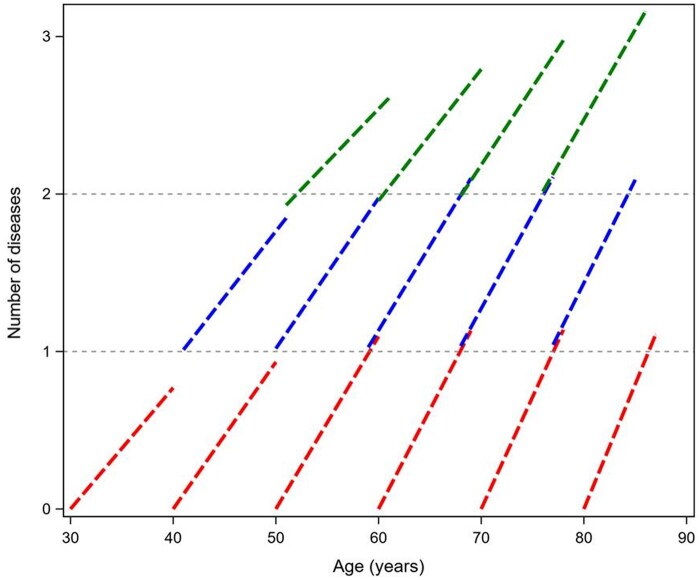
Longitudinal trajectories of multimorbidity accumulation according to different number of diseases (0, 1, or 2 diseases) and ages at the study entry. Red line: no diseases at baseline, *n* = 5593; blue line: 1 disease at baseline, *n* = 6054; green line: 2 diseases at baseline, *n* = 4127.

For the primary objective of our research, which was to investigate the relationship between duration of health expectancy and rate of accumulation of further diseases after the onset of the first condition, we needed to exclude participants who already had at least one disease at the study baseline. However, we questioned whether this sample selection could have affected the generalizability of our findings. Therefore, to address this issue, we ran an additional analysis using data from the eligible participants from the whole cohort to test whether the rate of multimorbidity accumulation was similar in participants who at the first HRS evaluation already had zero, one, or two diseases. Results from this additional analysis are represented in Figure 3, which illustrates the longitudinal trajectories of multimorbidity accumulation based on the baseline number of chronic diseases using data from participants from the three HRS cohorts with 0, 1, or 2 diseases at baseline (*N* = 5593, *N* = 6054, and *N* = 4127, respectively). The trajectories were plotted under the assumption that individuals who had (*n*) diseases at baseline and subsequently developed (*n* + 1) diseases during follow-up were comparable to those who had (*n* + 1) diseases from the outset. Our analysis revealed that within each age group, the rate of multimorbidity accumulation did not significantly differ based on the number of diseases reported at baseline. These findings suggest that the results from our selected sample of participants, who were healthy at baseline, are generalizable to the broader population.

## Discussion

Using longitudinal data from HRS participants who were free of nine chronic diseases at baseline but developed at least one during follow-up, we investigated the dynamic relationship between health expectancy—operationalized as the years of life free of chronic diseases—and the subsequent rate of accumulation of additional chronic conditions after the end of the disease-free period. Our findings indicate that both men and women who experienced their first disease at older ages tend to experience a faster accumulation of additional diseases compared to those with earlier onset. Additionally, we investigated the relationship between health expectancy, disease accumulation after the end of the disease-free period of life, and survival outcomes. We found that both a longer health expectancy and a slower rate of disease accumulation were significantly and independently associated with higher survival rates within our sample population. Notably, we found that the shorter the health expectancy, the stronger the association between the rate of disease accumulation after the detection of the first disease and mortality, suggesting that some substantial components of health deterioration in the old are not captured by the chronic diseases investigated in this study. Thus, the impact of the rate of diseases accumulation on mortality diminishes as individual’s age, potentially because advancing age itself becomes the dominant factor influencing mortality risk. Furthermore, we demonstrated that percentage of life in good health is positively associated with both health expectancy and the rate of accumulation of additional diseases after the onset of the first condition.

**Figure 4.**
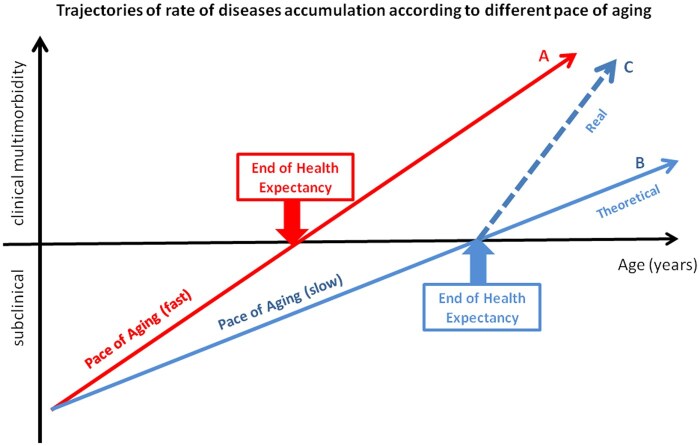
Trajectories of rate of chronic diseases accumulation according to different pace of aging.

We believe that the current work provides a significant contribution to the existing literature on a research theme that has been little explored. The positive association between health expectancy and the rate of accumulation of diseases is both surprising and logical at the same time. As represented in Figure 4, if we assume that longer health expectancy is due to a slower pace of aging, we will also expect that those individuals with longer health expectancy would experience a slower rate of multimorbidity accumulation after the detection of the first disease (line B) compared to those with a faster pace of aging (line A). Contrary to what was theoretically expected, our study demonstrated that individuals with longer health expectancy presented a higher rate of diseases accumulation after the onset of the first condition (line C). At a first sight, it is not surprising that individuals with longer health expectancy who start accumulating diseases at an older age may have the faster accumulation of multimorbidity observed here. Also, faster accumulation of diseases after longer health expectancy would suggest that increasing health expectancy may be followed by a proportionally faster decline in health, which would be consistent with the idea of “compression of morbidity.”^35^ Indeed, the fact that the speed of multimorbidity accumulation is a stronger predictor of mortality in individuals with shorter health expectancy compared to those with longer one suggests that aging “per se” rather than disease becomes the main driver of declining health at the time when individuals become frail as defined by Olshansky.^36^ These data these data indicate that interventions aimed at expanding health expectancy may lead to a compression of morbidity, ultimately reducing the number of years spent living with diseases and disabilities. Consistent with this hypothesis, we found that individuals with a longer health expectancy and a faster rate of multimorbidity accumulation after the end of health expectancy experience the highest percentage of life in good health.

While it is difficult to speculate on the underlying causes of these findings, our data indicate that developing the first chronic disease at older ages, compared to younger ages, leads to faster accumulation of additional diseases. We acknowledge that the timing of diagnosis can vary depending on additional factors, rather than just biological ones, such as individual socioeconomic status, access to healthcare, and diagnostic practices. In fact, once a person receives an initial diagnosis, the interaction with the healthcare system increases and it is possible that subsequent conditions are detected earlier due to increased and regular medical check-ups, but there is no reason to believe that this effect would be different between young and old age. Going back to the idea of biological aging, one plausible explanation may lay in the age-related decline of resilience across multiple systems. Specifically, individuals with a longer health expectancy experience a gradual, congruent decline in various resilience systems as they age. If none of these systems reaches the threshold of failure, overall health is maintained. However, when one system begins to fail, dysfunction reverberates on other bodily systems. This is because, at that point, there is little to no redundancy in the remaining systems, leading to a sudden loss of homeostasis throughout the entire organism. This condition was described by the American physician, poet, and novelist Oliver Wendell Holmes Sr. in the “*The Wonderful One-Hoss Shay*” that “*… went to pieces all at once.*”^37^

The geroscience hypothesis poses that interventions that slow down the pace of biological aging could delay the onset of age-related chronic diseases, thereby increasing health expectancy.^6^^,^^38^ Our findings suggest that increasing health expectancy could help achieve the goal of compressing morbidity. This will lead to fewer years spent living with chronic diseases (and likely also with disability), a reduced healthcare burden and costs, and an improved overall quality of life. Ultimately, these improvements would provide significant relief for individuals, their families, and the healthcare system.

Our study has several potential limitations. First, despite the availability of numerous instruments and metrics designed to assess multimorbidity for research and clinical applications, there is currently no universally accepted gold-standard definition. Considerable heterogeneity exists in both the criteria used to define chronic conditions and the classification schemes employed. As a result, there is no standardized list of conditions that meet the criteria for chronicity, and there is significant variability in the number and types of conditions included across studies.^39^ For a more standardized approach to develop an indicator of the number of chronic conditions, a conceptual model has been previously proposed, based on two related dimensions: (1) identifying and specifying chronic conditions of interest, and (2) understanding the structure of the data system of interest.^39^ Consistent with this approach, we operationalized multimorbidity as the count of chronic diseases ascertained longitudinally in the HRS. These diseases are common among older adults and strongly impact well-being. However, we cannot exclude that the selection of different diseases may have affected our findings. Furthermore, we focused on cumulative multimorbidity, expressed as diseases’ count. Diverse approaches to capture the burden of multimorbidity have been explored in literature. For example, previous authors developed a multimorbidity index weighted to physical functioning (Short-form-36 physical functioning scale) in three large cohorts of community-dwelling adults and validated it in the HRS participants.^40^ We acknowledge that the lack of information about the severity of the diseases may also represent an important limitation. Moreover, we acknowledge that there could be heterogeneity among the conditions included in our index about the impact on patient burden and risk of subsequent morbidity and mortality, which has not been addressed in the current analysis. Besides, we acknowledge that some chronic conditions that may be relevant for healthy longevity, for example, anemia and severe chronic kidney disease, have not been included in our cumulative multimorbidity index because information about their status was not available in the HRS Survey. Furthermore, our definition of chronic conditions, excluding cognitive impairment and depression, was based on self-reported diseases and did not include any functional measures. Different definitions of chronic conditions and metrics of multimorbidity should be tested in future studies to understand whether our findings are robust to such different definitions and operationalizations. Moreover, in the current analysis, we did not explore the association of health expectancy with the transition into multimorbidity and/or the sequence of acquisition of chronic diseases in such transition. Indeed, these would be a relevant topic for future research. Furthermore, to define health expectancy as years of life free of chronic diseases the ideal study design would be a life course approach,^41-44^ not available in HRS. Therefore, to calculate health expectancy in HRS participants, we selected individuals who were healthy (free of chronic diseases) at baseline and who developed at least one chronic condition over the follow-up. Participants who already had diseases at baseline (about 71.5% of the original sample population) were excluded because they did not have information about the time of onset of the first condition. Moreover, further 1319 individuals (about 23% of the disease-free participants) were additionally excluded because they did not develop any disease over the ­follow-up. Indeed, the selection process may affect the power of the study and the generalizability of the results. To partially address this issue, we ran an additional analysis that showed that the rate of multimorbidity accumulation was similar in HRS ­participants irrespective of their number of diseases at baseline (Figure 3). Certainly, further studies in larger and more diverse populations are needed to replicate and validate our results and confirm their generalizability. Specifically, we plan to replicate our analysis across different aging cohorts, using different metrics of multimorbidity and even including different sources of health information, including data from the public domain (ie, HRS-linked Medicare data). Finally, the most important limitation of this study is that we tested this hypothesis in an observational study, and we cannot exclude that the expansion of health expectancy following interventions will have the same results as anticipated by our observational study. Many scientists and Medical Societies are advocating that more investment should be dedicated to prevention through environmental, behavioral, and pharmacological interventions that target the enhancement of health in addition to the cure of disease which is most of the current focus. Geroscience, by studying and testing interventions that enhance biological resilience and slow down the accumulation of macromolecular damage with aging, may play an important role in this new emphasis on health and prevention.

## Supplementary Material

glaf164_Supplementary_Data
