# Supplementary material for: Dynamics of multimorbidity, health expectancy, and survival in middle aged and older individuals
Source: J Gerontol A Biol Sci Med Sci. 2025 Jul 29;80(9):glaf164. doi: 10.1093/gerona/glaf164 (PMC12401489; doi:10.1093/gerona/glaf164)
Supplement: glaf164_Supplementary_Data [file glaf164_supplementary_data.docx]

**Supplemental Materials**

**Figure S1. HRS Cohorts selection**

**Figure S2. Flowchart of the selection of the study population**

*All individuals with information on multimorbidity*

Early Baby Boomers

(N=4947)

War Babies

(N=2762)

Original HRS

(N=11966)

Overall sample (N=19675)

*Selection of individuals who were healthy (free of chronic diseases) at baseline*

Individuals free of chronic diseases at baseline (N=5593)

*Selection of individuals who developed at least one disease over the follow up*

Individuals with available information on health expectancy (N=4274)

*Selection of individuals aged 51 or older*

Individuals aged 51 or above (N=3511)

**Table S1. Characteristics of the study sample population (n=4274)**

| **Characteristics** | **Study Sample** |
| --- | --- |
| **Baseline Age, years**  **Mean (±sd)** | 55.1 (±6.3) |
| **Sex (men)**  **N (%)** | 1849 (43.3%) |
| **Ethnicity**   1. **Caucasian** 2. **African American** 3. **Others**   **N(%)** | 3536 (82.8%)  480 (11.2%)  254 (5.9%) |
| **Education (years)**  **Median (IQR)** | 13 (12-16) |
| **Baseline BMI, kg/m^2^**  **Mean (±sd)** | 26.5 (±4.6) |
| **Health expectancy (=years of life free of chronic diseases)**  **Mean (±sd)** | 61.3 (± 7.6) |
| **Rate of accumulation of additional diseases after the first onset,**  **N^ Diseases per year**  **Mean (±sd)** | 0.09 (±0.002) |
| **Follow up time, years**  **Mean (±sd)** | 9.4 (±5.4) |
| **Survival rate N (%)** | 3279 (76.7%) |

**Figure S3. Distribution of Age at onset of first chronic disease (=End of Health Expectancy) and Rate of accumulation of additional diseases (N=4274)**


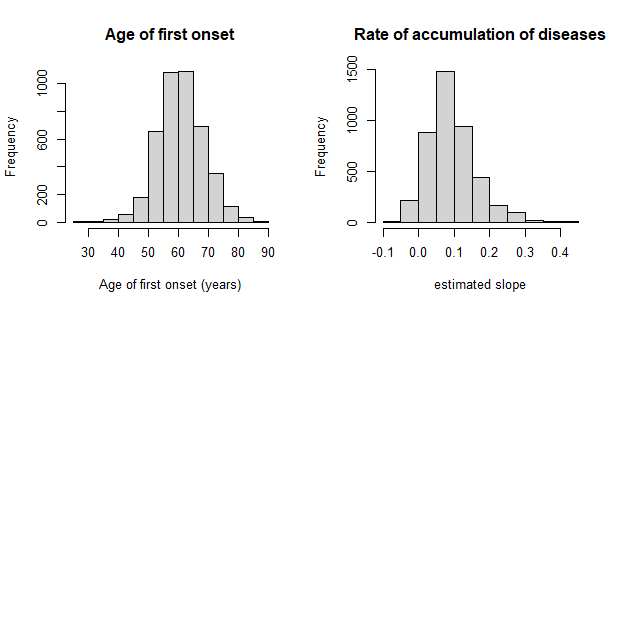


**Figure S4. Plot exploring the correlation between Age at onset of first chronic disease (years) and the rate of diseases accumulation (N=4274)**


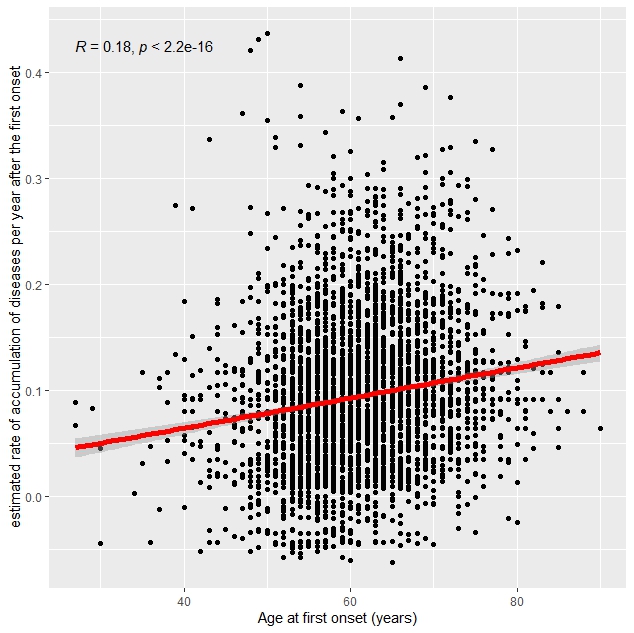


**Figure S5. Kaplan Meier Survival Curves according to different tertiles of age at onset of the first diseases (N=4274)**

**
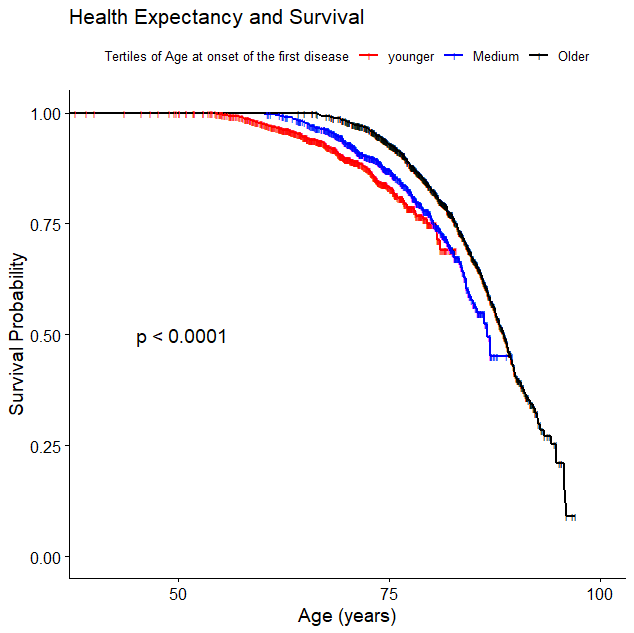
**

**Legend: Red line: younger tertile (=shorter health expectancy); Blue line: medium tertile; Black line: older tertile (= longer health expectancy)**

**Note: P value refers to the log-rank test**

**Figure S6. Kaplan Meier Survival Curves according to different tertiles of rate of diseases accumulation after the end of health expectancy (N=4274)**

**
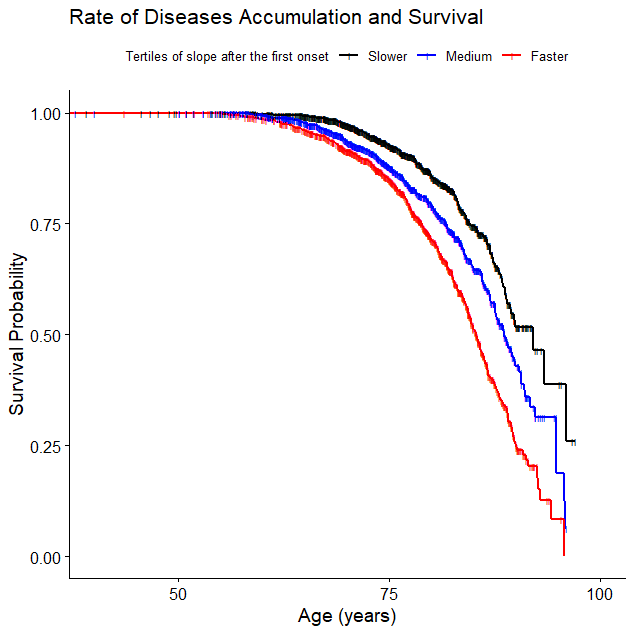
**

**Legend: Red line: faster tertile; Blue line: medium tertile; Black line: slower tertile**

**Note: P value refers to the log-rank test**
